# Three-Year Outcomes of Neoadjuvant Chemoimmunotherapy vs. Neoadjuvant Chemoradiotherapy in Resectable Esophageal Cancer: A Multicenter Retrospective Study

**DOI:** 10.3390/cancers18010155

**Published:** 2026-01-01

**Authors:** Shilong Deng, Xue Yan, Ying Peng, Lijun Zhu, Yongshi Shen, Wenmin Ying, Yuanji Xu, Zhichao Fu

**Affiliations:** 1 Department of Radiotherapy, Fuzong Clinical Medical College of Fujian Medical University, Fujian Medical University, Fuzhou 350025, China; dslong2014@fjmu.edu.cn (S.D.); yanxue20211118@163.com (X.Y.); doctorzhulijun@163.com (L.Z.); 2 Department of Radiotherapy, 900th Hospital of PLA Joint Logistic Support Force, Fuzhou 350025, China; 3 Department of Radiation Oncology, Clinical Oncology School of Fujian Medical University, Fujian Cancer Hospital, Fuzhou 350014, China; popyng@163.com (Y.P.); sys1014@fjmu.edu.cn (Y.S.); 4 Department of Radiotherapy, Fuding Hospital, Fuding 355200, China; ywm15080015210@163.com

**Keywords:** locally advanced resectable esophageal squamous cell carcinoma, neoadjuvant chemoradiotherapy, immunotherapy, survival

## Abstract

**Background**: Patients with locally advanced esophageal squamous cell carcinoma (LA-ESCC) have poor prognosis after surgery. Neoadjuvant chemoimmunotherapy (nCIT) and neoadjuvant chemoradiotherapy (nCRT) may improve outcomes, but their long-term efficacy remains unclear. **Methods**: This multicenter study analyzed LA-ESCC patients from three Chinese hospitals (2015–2024) who received nCIT or nCRT followed by surgery. Primary endpoint was 3-year overall survival (OS); secondary endpoints included objective response rate (ORR), pathologic complete response (pCR), disease-free survival (DFS), and adverse events. Propensity score matching balanced baseline characteristics. **Results**: Among 225 patients (87 nCRT, 138 nCIT), matched cohorts (87 per group) showed that nCRT had higher ORR (85.06% vs. 45.98%), T/N downstaging rates (78.16% vs. 58.62%; 85.06% vs. 45.98%), and pCR (37.90% vs. 14.90%) (all *p* < 0.01). After median follow-up (nCIT: 44.5 months; nCRT: 35.1 months), nCIT improved 3-year OS (75.90% vs. 55.60%) and DFS (66.40% vs. 47.30%) (*p* < 0.05). Subgroup analysis favored nCRT in N+ or non-cT4 disease. Clinical N stage independently predicted survival. **Conclusion**: nCIT demonstrates superior survival benefits in LA-ESCC, while nCRT may be more effective for N+ or non-cT4 patients. Further randomized trials are warranted.

## Simple Summary

Evidence regarding neoadjuvant chemoimmunotherapy (nCIT) or neoadjuvant chemoradiotherapy (nCRT) for resectable locally advanced esophageal squamous cell carcinoma (LA-ESCC) remains controversial. This study (*n* = 225) investigated the long-term efficacy and safety of nCIT versus nCRT in patients with LA-ESCC. The results suggest that nCIT can improve the long-term survival of patients with resectable esophageal cancer, whereas nCRT may provide greater benefits in patients with node-positive (N+) or non-cT4-stage disease. This study demonstrates the clinical efficacy and safety of nCIT in patients with LA-ESCC.

## 1. Introduction

Esophageal cancer is one of the most common cancers globally, ranking seventh in incidence and sixth in mortality among all cancers [1]. Esophageal squamous cell carcinoma (ESCC) is the predominant subtype of esophageal cancer in Asia [2], accounting for over 84% of all esophageal cancer cases [3]. In the early stages of esophageal cancer, patients often remain asymptomatic, leading to the majority being diagnosed at a locally advanced stage. Currently, esophagectomy is the primary treatment for patients with resectable locally advanced ESCC (LA-ESCC). However, surgery alone often results in high recurrence rates and poor survival outcomes [3].

There is growing evidence that neoadjuvant therapy can improve survival in patients with LA-ESCC. The CROSS clinical trial demonstrated that neoadjuvant chemoradiotherapy (nCRT) followed by surgery significantly improved overall survival (OS) in patients with LA-ESCC compared to surgery alone [4,5]. Similarly, the JCOG9907 clinical study compared the efficacy and safety of neoadjuvant chemotherapy (nCT) to adjuvant chemotherapy in treating LA-ESCC, finding that nCT significantly enhanced OS with an acceptable safety profile [6]. Immune checkpoint inhibitors (ICIs), such as PD-1 and PD-L1 inhibitors, have demonstrated efficacy in treating advanced ESCC and other cancers [7,8,9]. Additionally, several preclinical studies have highlighted the synergistic effects of combining ICIs with chemotherapy or radiotherapy [10,11]. Despite these advancements, there is still a limited number of studies comparing the efficacy of nCRT versus neoadjuvant chemotherapy combined with immunotherapy (nCIT) in resectable LA-ESCC [12,13,14].

Therefore, this study aimed to compare the treatment responses and long-term survival outcomes of patients with resectable LA-ESCC who received nCRT versus nCIT followed by esophagectomy.

## 2. Methods

### 2.1. Methods and Patients

This retrospective study included patients with LA-ESCC who received either nCIT or nCRT, followed by esophagectomy, at three hospitals (900th Hospital of PLA Joint Logistic Support Force, Fujian Cancer Hospital, and Quanzhou Guangqian Hospital) in China between November 2015 and January 2024. The inclusion criteria were as follows: (1) age > 18 years old; (2) ECOG score of 0 to 1; (3) thoracic ESCC with clinical stage from T2-4N0M0 or TanyN+M0 based on AJCC 8th; (4) completed neoadjuvant therapy followed by surgery; (5) postoperative pathologic diagnosis of squamous cell carcinoma; (6) No history of any antitumor therapy, such as targeted therapy or immunotherapy; and (7) Availability of complete clinical data and survival or follow-up information. The exclusion criteria were as follows: (1) postoperative pathological diagnosis of non-squamous cell carcinoma; (2) incomplete neoadjuvant therapy or failure to undergo transthoracic esophagectomy following neoadjuvant therapy; (3) receipt of radical radiation therapy; (4) missing clinical data; and (5) presence of other concurrent malignant tumors. The study was approved by institutional ethics board of the 900th Hospital of PLA Joint Logistic Support Force (No. 2024-032) and individual consent for this retrospective analysis was waived.

### 2.2. Treatments

Patients in the nCRT group received concurrent chemoradiotherapy. The radiotherapy regimen consisted of 40–50 Gy, delivered in 20–25 fractions, with 5 fractions per week. Concurrent chemotherapy included two options: (1) paclitaxel (45–60 mg/m^2^) combined with cisplatin (20–25 mg/m^2^) weekly, or (2) cisplatin (30 mg/m^2^) combined with capecitabine (800 mg/m^2^) weekly. Patients in the nCIT group received immunotherapy in combination with concurrent taxane plus platinum (TP) chemotherapy. Immunotherapy agents included toripalimab (240 mg every 3 weeks), camrelizumab (200 mg every 3 weeks), pembrolizumab (200 mg every 3 weeks), sintilimab (200 mg every 3 weeks), or tislelizumab (200 mg every 3 weeks). The TP chemotherapy regimen included albumin-bound paclitaxel (175 mg/m^2^) or docetaxel (70 mg/m^2^) combined with cisplatin (75 mg/m^2^) or nedaplatin (75 mg/m^2^), administered every 3 weeks. After completing neoadjuvant therapy, all patients in both the nCIT and nCRT groups underwent minimally invasive McKeown esophagectomy.

As part of their routine clinical management, all patients received one of two standard neoadjuvant treatment regimens prior to surgery, independent of this study. The treatments were administered by the patients’ clinical care teams based on established hospital protocols, and this research did not influence or administer any interventions.

### 2.3. Endpoints and Assessments

The primary endpoint was 3-year OS rate. Second endpoints included pathologic complete response (pCR), major pathological response (MPR), objective response rate (ORR), disease- free survival (DFS) and treatment-related adverse events (TRAEs). Adverse events (AEs) were assessed according to the Common Terminology Criteria for Adverse Events, version 5.0 (CTCAE v5.0). MPR was defined as ≤10% residual viable tumor cells in the resected tumor bed, evaluated by H&E-stained slides [15]. pCR was defined as the absence of viable tumor cells in both the primary tumor site and resected lymph nodes [16]. OS was defined as the time from treatment to death from any cause, while DFS referred to the time from treatment to disease progression, recurrence at any site, or death from any cause [17].

### 2.4. Statistical Analysis

Categorical variables were compared between the two groups using the chi-square test or Fisher’s exact test, and numerical variables were compared using the independent *t*-test. Propensity score matching (PSM) was applied to minimize baseline differences between the nCIT and nCRT groups, adjusting for measured confounders [18]. Univariate and multivariate Cox regression analyses were conducted to identify independent prognostic factors for OS and DFS in patients with locally advanced esophageal squamous cell carcinoma (LA-ESCC). Kaplan–Meier survival curves and log-rank tests were used to compare OS and DFS between the two groups. Statistical analyses were performed using R software (v4.4.1) and SPSS (v27.0), with a significance threshold set at a *p*-value < 0.05.

## 3. Results

### 3.1. Patient Clinical Characteristics

**Figure 1:**
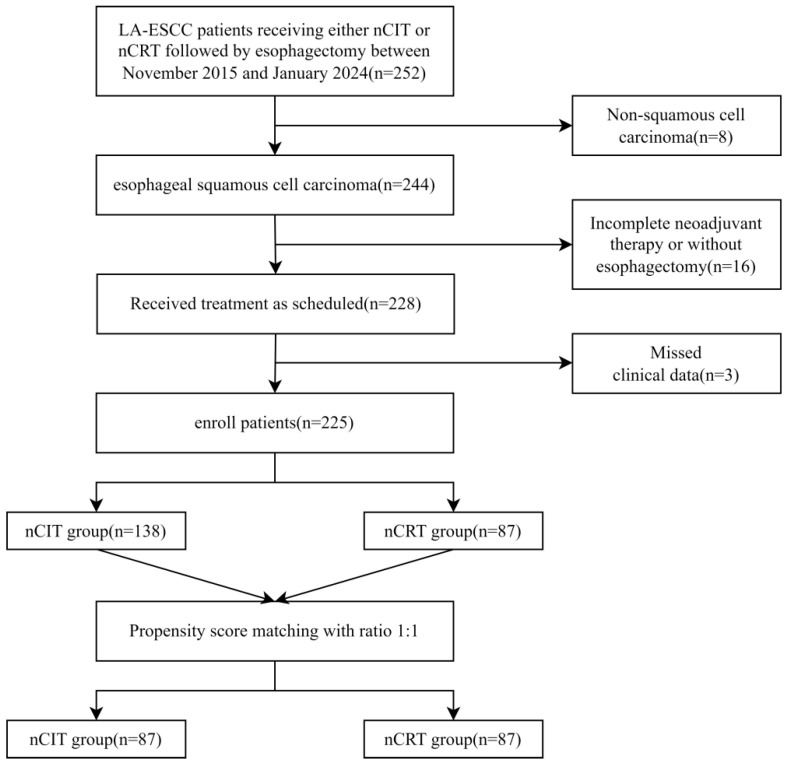
Patient flowchart. LA-ESCC: Locally advanced esophageal squamous cell carcinoma; nCIT: Neoadjuvant chemotherapy plus immunotherapy; nCRT: Neoadjuvant chemoradiotherapy.

A total of 225 patients were included in this study, including 138 patients who received nCIT and 87 patients who received conventional nCRT as shown in Figure 1. The median follow-up duration was 44.5 months for the nCIT group and 35.1 months for the nCRT group.

Baseline characteristics were presented in Table 1. The two groups differed significantly in age, smoking status, tumor location, and clinical T stage, but there were no significant differences in sex, alcohol history, clinical N stage, or clinical stage. To address baseline imbalances, a 1:1 case–control analysis was performed using PSM. After PSM adjustment, baseline characteristics were well-balanced between the two treatment groups, as shown in Table 2. Each group consisted of 87 patients following matching.

Data on baseline patient characteristics, including smoking and drinking history, were retrieved from electronic medical records. A ‘smoker’ was defined as a patient with a documented history of regular cigarette use (≥1 cigarette/day) for ≥6 months. A ‘drinker’ was defined as a patient with a documented history of regular alcohol consumption (≥1 time/week) for ≥6 months.

### 3.2. Efficacy Results

As presented in Table 3, the nCRT group showed significantly better outcomes than the nCIT group in terms of ORR (85.06% vs. 45.98%, *p* < 0.001), postoperative T stage (78.16% vs. 58.62%, *p* = 0.006) N stage descending rate (85.06% vs. 45.98%, *p* < 0.001) as well as the pCR rate (37.9% vs. 14.9%, *p* < 0.001).

**Figure 2:**
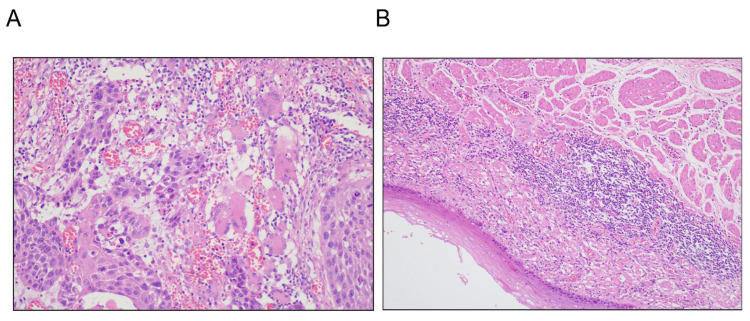
Representative histopathological images of tumor response. (**A**) Pathologic complete response (pCR), defined as the absence of any residual viable tumor cells. (**B**) Major pathological response (MPR), defined as ≤10% residual viable tumor cells.

To histologically validate the treatment responses observed in this cohort, representative hematoxylin and eosin (H&E)-stained sections from resected specimens were examined. As illustrated in Figure 2, a spectrum of pathologic responses was achieved following neoadjuvant therapy. Figure 2A demonstrates a pathologic complete response (pCR), characterized by the complete absence of any residual viable tumor cells, with only fibrotic stroma and inflammatory infiltrate remaining. In contrast, Figure 2B shows a major pathological response (MPR), defined as ≤10% residual viable tumor cells amidst extensive treatment-induced regression. These representative images provide concrete histopathological evidence for the response criteria (pCR and MPR) that were central to our survival analysis.

**Figure 3:**
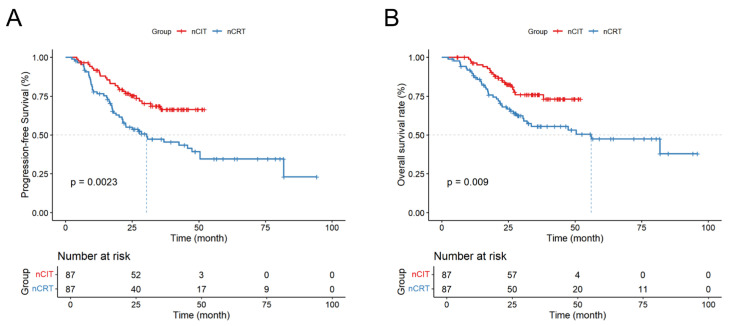
Survival analysis for LA-ESCC patients in the nCIT group and the nCRT group. (**A**) Kaplan–Meier plot of disease free survival; (**B**) Kaplan–Meier plot of overall survival.

**Figure 4:**
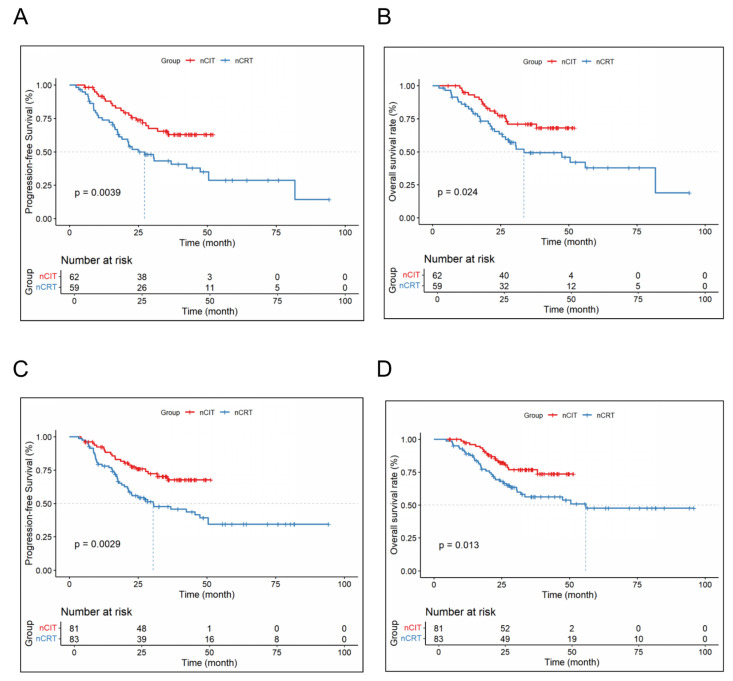
Subgroup survival analysis for lymph node-positive and non-cT4-Stage LA-ESCC patients. (**A**) Disease-free survival and (**B**) overall survival in LA-ESCC patients with lymph node-positive status. (**C**) Disease-free survival and (**D**) overall survival in LA-ESCC patients with non-cT4 stage.

**Figure 5:**
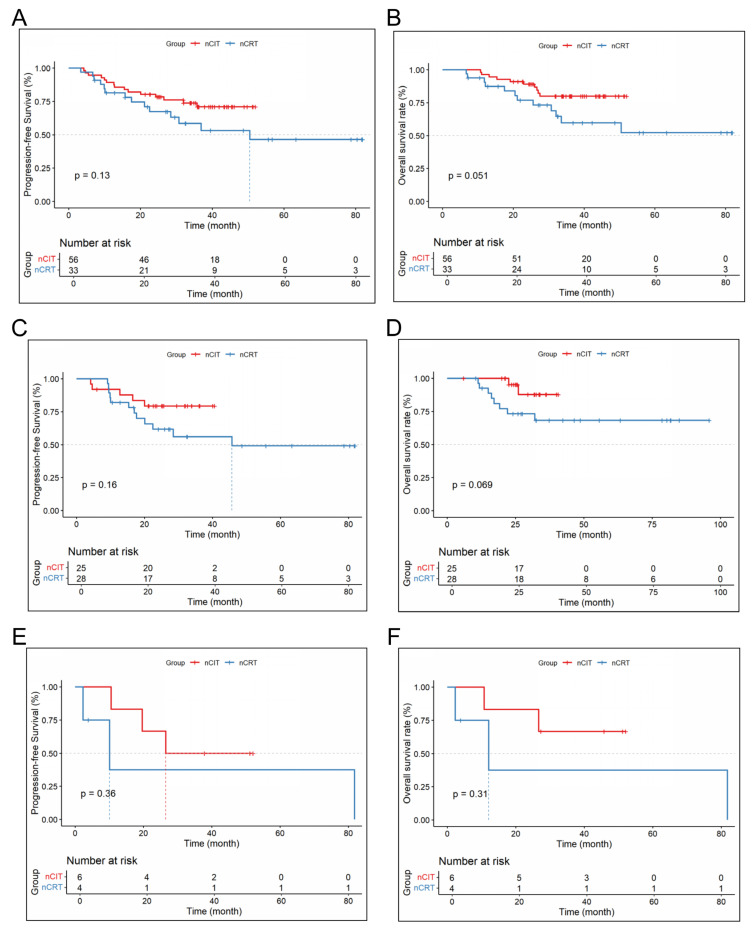
Subgroup survival analysis for LA-ESCC patients by pathological status. (**A**) Disease-Free survival and (**B**) overall survival in patients with pCR. (**C**) Disease-free survival and (**D**) overall survival in lymph node-negative patients. (**E**) Disease-free survival and (**F**) overall survival in patients with clinical T4 stage (cT4).

The 1-, 2-, and 3-year DFS and OS rates for the nCRT and nCIT are shown in Table 4. Additionally, nCIT group had longer OS and DFS compared to the nCRT group (*p* < 0.05) (Figure 3). Subgroup analysis revealed that patients with LA-ESCC and clinical N+ or non-cT4 stage in the nCRT group had significantly longer mOS and mDFS (Figure 4). However, for patients with LA-ESCC and clinical N- or cT4 stage, there were no significant differences in OS and DFS between nCIT group and nCRT group (Figure 5). In addition, among patients with pCR, there was no significant difference in OS and DFS between the two treatment groups (Figure 5).

**Figure 6:**
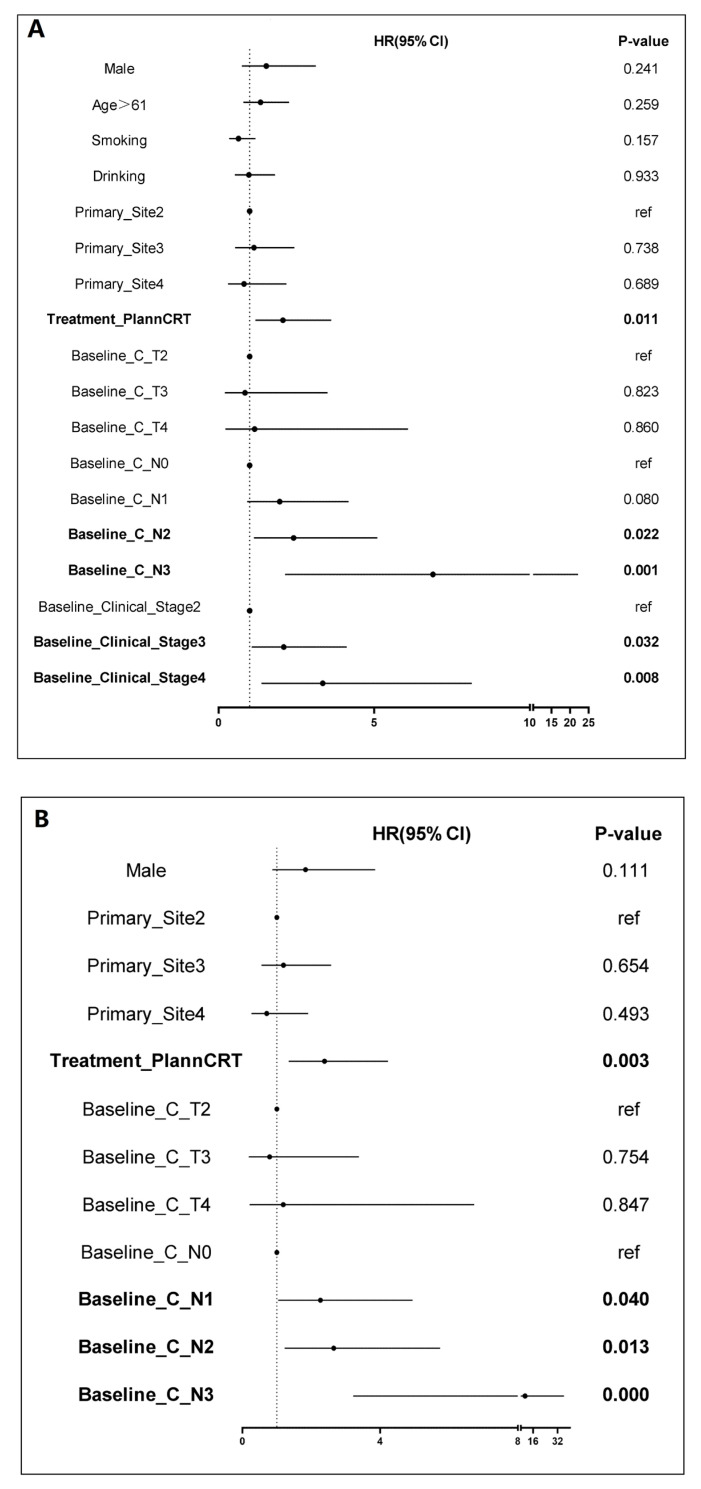
Univariate and multivariate cox regression analysis of overall survival for LA-ESCC Patients. (**A**) Univariate Cox regression analysis; (**B**) Multivariate Cox regression analysis.

**Figure 7:**
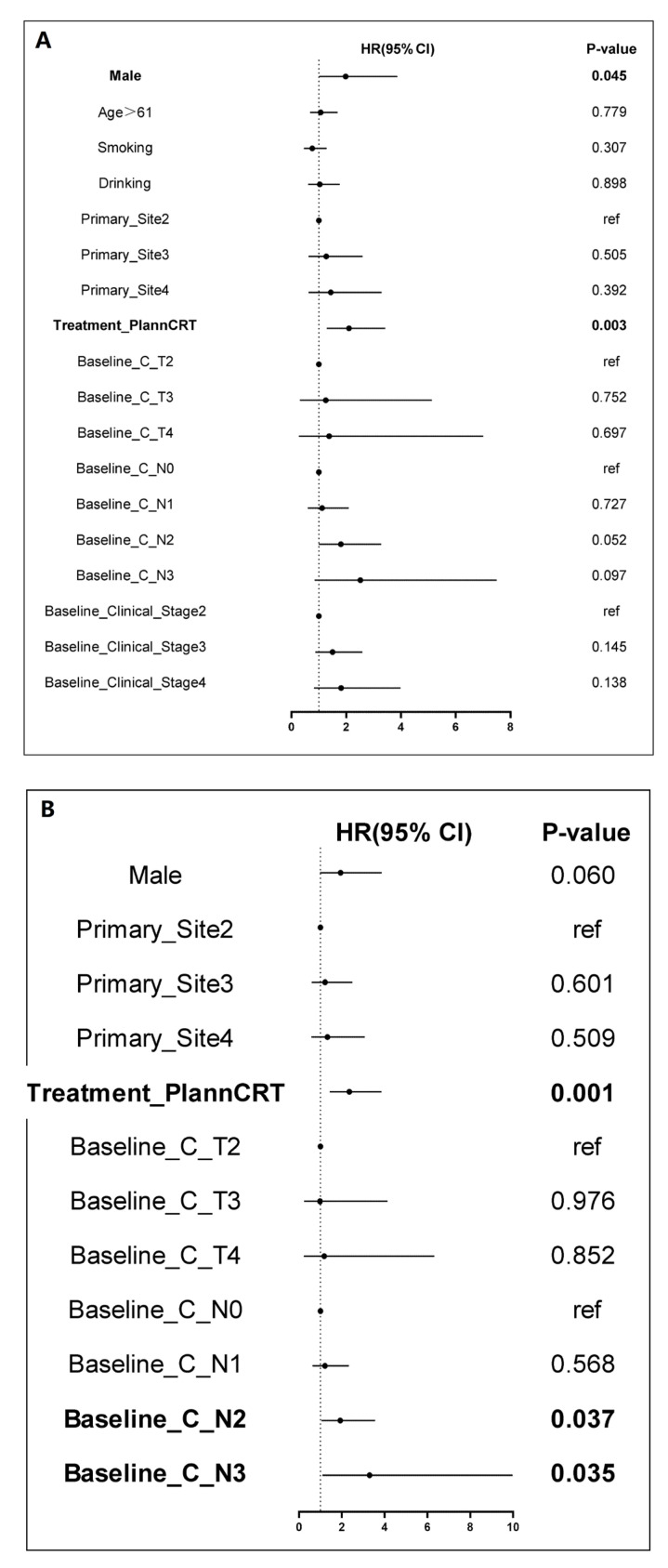
Univariate and multivariate Cox regression analysis of disease-free survival for LA-ESCC patients. (**A**) Univariate Cox regression analysis; (**B**) Multivariate Cox regression analysis.

Univariate and multivariate Cox regression analyses identified clinical N stage as an independent prognostic factor for OS and DFS. Specifically, for OS, the hazards ratios (HR) for N1, N2, and N3 were 2.27 (95% CI = 1.04–4.94, *p* = 0.04), 2.65 (95% CI = 1.23–5.74, *p* = 0.013), and 10.79 (95% CI = 3.22–36.17, *p* < 0.001), respectively, as shown in Figure 6. For DFS, the HR for N2 was 1.93 (95% CI = 1.04–3.56, *p* = 0.037), and for N3, the HR was 3.30 (95% CI = 1.09–9.98, *p* = 0.035) as shown in Figure 7. These findings suggest that LA-ESCC patients with clinical N2 or N3 stage are at higher risk for tumor progression and have poorer prognosis.

### 3.3. Safety Results

AEs in this study were defined as complications occurring from the initiation of neoadjuvant therapy through one week after surgery completion. As shown in Table 5, after PSM, no statistically significant differences were observed in the incidence of myelosuppression of Grade ≥ 3 (16.09% vs. 17.24%), liver dysfunction of Grade ≥ 3 (2.30% vs. 0%), vomiting of Grade ≥ 3 (6.70% vs. 2.30%), pneumonia (36.78% vs. 24.14%), or esophageal fistula (1.15% vs. 3.45%) between the nCRT and nCIT groups (*p* > 0.05).

## 4. Discussion

nCIT, as a novel neoadjuvant therapeutic approach, has been demonstrated to exhibit definitive efficacy in various solid tumors, such as non-small cell lung cancer and triple-negative breast cancer [19,20]. In the first reported multicenter clinical study, we observed that patients with LA-ESCC treated with nCIT followed by esophagectomy demonstrated better OS and DFS compared to those receiving nCRT followed by surgery. A retrospective study by Yu YK et al. which analyzed the efficacy of neoadjuvant therapy in 202 patients with LA-ESCC (81 receiving nCIT and 121 receiving nCRT), found that after adjustment for inverse probability of treatment weighting, the nCIT group had superior 3-year OS (91.70% vs. 79.80%; *p* = 0.032) and 3-year DFS (87.40% vs. 72.80%; *p* = 0.039) compared to the nCRT group, which was similar to the results of our study [21]. However, contrasting findings have been reported in other single-center studies. Zhao et al. reported that the nCRT group had significantly superior DFS and OS compared to the nCIT group (12-month DFS: 94.30% vs. 81.80%, *p* = 0.006; 12-month OS: 100.00% vs. 95.40%, *p* = 0.032) [22]. These mixed results underscore the need for further research to compare the efficacy of nCRT and nCIT in LA-ESCC.

Our study’s multivariate Cox regression analysis identified clinical N stage as an independent prognostic factor for both DFS and OS (*p* < 0.05). With data from multiple centers and long-term follow-up, our research provides a robust comparison of these two treatment modalities, highlighting the potential long-term benefits of tumor immunotherapy. Subgroup analysis revealed that patients with N-positive disease had significantly longer DFS and OS in the nCIT group (*p* < 0.05), suggesting that nCIT may offer particular advantages for LA-ESCC patients with N-positive status. Patients with lymph node-positive status often have undetectable metastases in the bloodstream at the time of diagnosis [23]. Compared to the localized effects of radiotherapy, immunotherapy may reduce recurrence in this patient population through its systemic therapeutic effects.

A retrospective study comparing the feasibility and safety of nCRT plus surgery versus nCIT plus surgery in 64 patients with resectable LA-ESCC found that the nCRT group had better pathological responses, with a pCR rate of 43.80% vs. 18.80% and a MPR rate of 71.90% vs. 34.40% compared to the nCIT group [24]. Similarly, our study showed that the nCRT group had a higher pCR rate (37.90% vs. 14.90%, *p* < 0.001). Furthermore, Another retrospective cohort study involving 44 patients (23 in the nCRT group and 21 in the nCIT group) found no significant difference in adverse event rates or post-surgery pathological remission rates between the two groups [25], which contrasts with our findings in terms of pathological response, likely due to retrospective biases and small sample size. Previous study have shown that patients achieving pCR often experience prolonged DFS and OS [26]. However, A meta-analysis indicated that patients with early-stage triple-negative breast cancer who achieved pathological complete response (defined as ypT0, ypN0 or ypT0/is ypN0) demonstrated improved survival rates [27]. The KEYNOTE-585 study also have shown that combining immunotherapy with chemotherapy increased the pCR rate by 10% compared to the chemotherapy-alone group, but neither EFS nor OS reached the predefined endpoints [28]. It is important to recognize that pCR does not always equate to optimal outcomes. Neoadjuvant therapies can cause adverse effects, such as cardiotoxicity, which may diminish patients’ quality of life. Additionally, even in cases where pCR is achieved, micrometastatic disease or minimal residual cancer may persist, potentially leading to recurrence or metastasis and, ultimately, treatment failure [29]. Consequently, while pCR rates are valuable, greater emphasis should be placed on DFS and OS as comprehensive measures of therapeutic success [30].

This study has several limitations. First, as a retrospective analysis, the results may still be subject to inherent biases despite the application of PSM to mitigate them. Additionally, the relatively small sample size limits the robustness of the findings, highlighting the need for larger studies. Furthermore, the heterogeneity in specific regimens within each treatment group (e.g., different immunotherapeutic agents and chemotherapy backbones) represents a potential source of confounding, while the absence of biomarker data (e.g., PD-L1, TMB) precludes analyses to identify predictive biomarkers of response. Finally, the follow-up period is limited, requiring longer follow-up to more accurately assess long-term survival outcomes. We plan to continue monitoring patient survival and update the findings in future studies.

## 5. Conclusions

Neoadjuvant chemoimmunotherapy followed by surgery is well tolerated and effective treatment option for locally advanced resectable esophageal squamous cell carcinoma, especially for patients with lymph node-positive disease.

## Figures and Tables

**Table 1:** Baseline characteristics.

Baseline Feature, *n* (%)	nCRT (*n* = 87)	nCIT (*n* = 138)	*p*
Sex			0.927
male	70 (80.5)	113 (81.9)	
female	17 (19.5)	25 (18.1)	
Age (years old)			0.047
≤61	53 (60.9)	64 (46.4)	
>61	34 (39.1)	74 (53.6)	
Smoking			0.003
no	65 (74.7)	75 (54.3)	
yes	22 (25.3)	63 (45.7)	
Drinking			0.153
no	70 (80.5)	98 (71.0)	
yes	17 (19.5)	40 (29.0)	
Tumor site			0.031
upper throacic segment	12 (13.8)	16 (11.6)	
middle thoracic segment	64 (73.6)	84 (60.9)	
lower thoracic segment	11 (12.6)	38 (27.5)	
Clinical T stage			0.008
T1	0 (0.0)	2 (1.4)	
T2	3 (3.4)	20 (14.5)	
T3	79 (90.8)	100 (72.5)	
T4	5 (5.7)	16 (11.6)	
Clinical N stage			0.131
N0	28 (32.2)	32 (23.2)	
N1	31 (35.6)	40 (29.0)	
N2	25 (28.7)	57 (41.3)	
N3	3 (3.4)	9 (6.5)	
Clinical stage			0.174
I	0 (0.0)	1 (0.7)	
II	30 (34.5)	33 (23.9)	
III	49 (56.3)	81 (58.7)	
IV	8 (9.2)	23 (16.7)	

nCIT: Neoadjuvant chemotherapy plus immunotherapy; nCRT: Neoadjuvant chemoradiotherapy.

**Table 2:** Baseline characteristics after PSM.

Baseline Feature, *n* (%)	nCRT (*n* = 87)	nCIT (*n* = 87)	*p*
Sex			0.852
male	70 (80.5)	68 (78.2)	
female	17 (19.5)	19 (21.8)	
Age (years old)			0.285
≤61	53 (60.9)	45 (51.7)	
>61	34 (39.1)	42 (48.3)	
Smoking			0.318
no	65 (74.7)	58 (66.7)	
yes	22 (25.3)	29 (33.3)	
Drinking			0.582
no	70 (80.5)	66 (75.9)	
yes	17 (19.5)	21 (24.1)	
Tumor site			0.465
upper thoracic segment	12 (13.8)	11 (12.6)	
middle thoracic segment	64 (73.6)	59 (67.8)	
lower thoracic segment	11 (12.6)	17 (19.5)	
Clinical T stage			0.953
T2	3 (3.4)	3 (3.4)	
T3	79 (90.8)	78 (89.7)	
T4	5 (5.7)	6 (6.9)	
Clinical N stage			0.875
N0	28 (32.2)	25 (28.7)	
N1	31 (35.6)	29 (33.3)	
N2	25 (28.7)	30 (34.5)	
N3	3 (3.4)	3 (3.4)	
Clinical stage			0.933
II	30 (34.5)	28 (32.2)	
III	49 (56.3)	50 (57.5)	
IV	8 (9.2)	9 (10.3)	

nCIT: Neoadjuvant chemotherapy plus immunotherapy; nCRT: Neoadjuvant chemoradiotherapy; PSM: Propensity score matching.

**Table 3:** Treatment responses of the two groups.

*n* (%)	nCRT (*n* = 87)	nCIT (*n* = 87)	*p*
PR/CR			<0.001
yes	74 (85.1)	40 (46.0)	
no	13 (14.9)	47 (54.0)	
pCR			<0.001
yes	33 (37.9)	13 (14.9)	
no	54 (62.1)	74 (85.1)	
MPR			0.034
yes	51 (58.6)	37 (42.5)	
no	36 (41.4)	50 (57.5)	
Postoperative T stage descending			0.006
yes	68 (78.2)	51 (58.6)	
no	19 (21.8)	36 (41.4)	
Postoperative N stage descending			<0.001
yes	74 (85.1)	40 (46.0)	
no	13 (14.9)	47 (54.0)	

nCIT: Neoadjuvant chemotherapy plus immunotherapy; nCRT: Neoadjuvant chemoradiotherapy; PR: Partial response; CR: Complete response; pCR: Pathologic complete response; MPR: Major pathological response.

**Table 4:** Long-term survival outcomes of the two groups.

	nCIT (%, *n*/N)	nCRT (%, *n*/N)
1-year DFS	91.72% (80/87)	76.55% (67/87)
2-year DFS	76.73% (67/87)	55.09% (48/87)
3-year DFS	66.35% (58/87)	47.32% (41/87)
1-year OS	96.43% (84/87)	88.27% (77/87)
2-year OS	82.49% (72/87)	68.03% (59/87)
3-year OS	75.89% (66/87)	55.57% (48/87)

nCIT: Neoadjuvant chemotherapy plus immunotherapy; nCRT: Neoadjuvant chemoradiotherapy; DFS: Disease-free survival; OS: Overall survival.

**Table 5:** Treatment-related adverse events post neoadjuvant therapy.

TRAEs *n* (%)	nCRT (*n* = 87)	nCIT (*n* = 87)	*p*
Myelosuppression			0.839
Grade 1/2	73 (83.9)	72 (82.8)	
Grade ≥ 3	14 (16.1)	15 (17.2)	
Liver dysfunction			0.497
Grade 1/2	85 (97.7)	87 (1)	
Grade ≥ 3	2 (2.3)	0	
Vomiting			0.278
Grade 1/2	81 (93.1)	85 (97.7)	
Grade ≥ 3	6 (6.9)	2 (2.3)	
Pneumonia			0.070
yes	32 (36.8)	21 (24.1)	
no	55 (63.2)	66 (75.9)	
Esophageal fistula			0.621
yes	1 (1.1)	3 (3.4)	
no	86 (98.9)	84 (96.6)	

TRAEs: Treatment-related adverse events; nCIT: Neoadjuvant chemotherapy plus immunotherapy; nCRT: Neoadjuvant chemoradiotherapy.

## Data Availability

The raw data from this study are not publicly available to protect patient privacy. For access to the research data, please contact the corresponding author.

## Abbreviations

The following abbreviations are used in this manuscript:

LA-ESCC	Locally advanced esophageal squamous cell carcinoma
ESCC	Esophageal squamous cell carcinoma
nCRT	Neoadjuvant chemoradiotherapy
nCIT	Neoadjuvant chemotherapy plus immunotherapy
OS	Overall survival
PFS	Progression-free survival
DFS	Disease-free survival
pCR	Pathologic complete response
ORR	Objective response rate
MPR	Major pathological response
PSM	Propensity score matching
TP	Taxane plus platinum
TRAEs	Treatment-related adverse events
AEs	Adverse events
PD-L1	Programmed death-ligand 1
TMB	Tumor mutational burden
